# Pilot study of ^89^Zr-bevacizumab positron emission tomography in patients with advanced non-small cell lung cancer

**DOI:** 10.1186/s13550-014-0035-5

**Published:** 2014-08-02

**Authors:** Idris Bahce, Marc C Huisman, Eline E Verwer, Rogier Ooijevaar, Firdaouss Boutkourt, Danielle J Vugts, Guus AMS van Dongen, Ronald Boellaard, Egbert F Smit

**Affiliations:** Department of Pulmonary Diseases, VU University Medical Center, De Boelelaan 1117, Amsterdam, 1081HV The Netherlands; Department of Radiology & Nuclear Medicine, VU University Medical Center, De Boelelaan 1117, Amsterdam, 1081HV The Netherlands; Department of Otolaryngology/Head and Neck Surgery, VU University Medical Center, De Boelelaan 1117, Amsterdam, 1081HV The Netherlands

**Keywords:** NSCLC, VEGF, Immuno-PET, 89Zr-bevacizumab

## Abstract

**Background:**

The aim of this pilot study was to evaluate whether the uptake of ^89^Zr-bevacizumab in non-small cell lung cancer (NSCLC) tumors could be visualized and quantified. The correlation between tumor ^89^Zr-bevacizumab uptake and tumor response to antitumor therapy with a bevacizumab-based regimen was explored.

**Methods:**

Seven NSCLC patients underwent static PET scans at days 4 and 7 after injection of 36.4 ± 0.9 MBq (mean ± SD) ^89^Zr-bevacizumab, prior to commencing carboplatin-paclitaxel-bevacizumab chemotherapy (CPB). Overall survival (OS) and progression-free survival (PFS) to CPB followed by bevacizumab maintenance therapy was correlated to tumor tracer uptake, quantified using peak standardized uptake values (SUV_peak_).

**Results:**

Zr-bevacizumab uptake (SUV_peak_) was approximately four times higher in tumor tissues (primary tumor and metastases) than in non-tumor tissues (healthy muscle, lung, and fat) on days 4 and 7. A positive trend but no significant correlation could be found between SUV_peak_ and OS or PFS.

**Conclusions:**

This pilot study shows that ^89^Zr-bevacizumab PET imaging in NSCLC is feasible. Further investigation to validate this technique as a predictive biomarker for selecting patients for bevacizumab treatment is warranted.

**Electronic supplementary material:**

The online version of this article (doi:10.1186/s13550-014-0035-5) contains supplementary material, which is available to authorized users.

## Background

Vascular endothelial growth factor (VEGF) is an important mediator in non-tumoral and tumoral angiogenesis. VEGF-A, one of the five members of the VEGF family and generally referred to as VEGF, is overexpressed in many tumors [1],[2]. In response to hypoxia, cells of mesenchymal, stromal, and epithelial origin (e.g., myocytes, blood platelets, and stromal cells in tumors) show paracrine secretion of VEGF-A. This increases VEGF-A concentrations locally in the tumor, causing a strong pro-angiogenic stimulus. The binding of VEGF-A to VEGF receptor subtypes 1 and 2 (VEGFR1 and VEGFR2), both transmembrane monomers expressed on vascular endothelial cells, causes dimerization of the VEGFR1/2 monomers, leading to the activation of intracellular pro-angiogenic signaling pathways [3]-[6]. Also, non-angiogenic effects of increased VEGF-A have been described [3],[7],[8].

The addition of bevacizumab, a monoclonal antibody directed against VEGF-A, to cytotoxic chemotherapy improves tumor response rates and provides a survival advantage over chemotherapy alone in several malignancies [9]-[12]. A phase III study showed a survival benefit for patients with non-small cell lung cancer (NSCLC) treated with carboplatin-paclitaxel-bevacizumab compared with chemotherapy alone [13]. However, on an individual basis, it is unclear who would benefit from bevacizumab therapy, as predictive markers are lacking, despite a large body of clinical research available to date.

Imaging tumor uptake of radiolabeled bevacizumab in patients using positron emission tomography (PET) may provide such a predictive marker, as PET imaging enables *in vivo* monitoring of physiological processes. To date, several clinical studies have been performed using antibodies with ^89^Zr labeling. In these studies, positive image contrast is observed, and its relation to therapy response discussed [14]-[17]. The aim of this pilot study was to evaluate whether ^89^Zr-bevacizumab enables visualization of NSCLC tumors and to assess the ranges of tumor-to-background ratios and standardized uptake values. Additionally, the correlation between ^89^Zr-bevacizumab uptake and tumor response following treatment with a bevacizumab-based regimen was assessed.

## Methods

### Study design

#### Patients

In this prospective pilot study, patients with stage IV adenocarcinoma of the lung who were scheduled for combined carboplatin-paclitaxel-bevacizumab treatment were enrolled. Inclusion criteria were histologic diagnosis of non-squamous NSCLC, age of 18 years or older, performance status of 0 to 2 (WHO), a life expectancy of at least 12 weeks, and presence of at least one NSCLC lesion within the chest of at least 1.5-cm diameter as measured by computed tomography (CT). Exclusion criteria included claustrophobia, pregnancy, lactation, and use of concurrent or previous treatment with anticancer drugs within 30 days prior to scanning. The patients gave informed consent, and this study was approved by the Medical Ethics Review Committee of the VU University Medical Center.

#### Imaging

Prior to the start of therapy, the patients underwent PET/CT scans using [^18^ F]fluorodeoxyglucose (FDG) and ^89^Zr-bevacizumab.

#### Therapy

As scheduled prior to inclusion in this study, the patients received treatment with carboplatin (area under the concentration-time curve of 6.0 mg/mL/min)-paclitaxel (200 mg/m^2^ of body surface area)-bevacizumab (15 mg/kg) and continuation maintenance bevacizumab upon non-progression after 4 cycles. Chemotherapy was given every 21 days until disease progression or unacceptable toxicity [13].

### Preparation of ^89^Zr-bevacizumab

Bevacizumab was labeled with zirconium-89 using *N*-succinyl-desferrioxamine (*N*-suc-Df) as described previously [18],[19]. All procedures were performed under aseptic conditions in a shielded laminar flow cabinet. In short, desferrioxamine (Desferal (Df), Novartis, Basel, Switzerland) was converted to *N*-succinyl-Df. Next, the hydroxamate groups were blocked with iron, and the succinic acid group converted to its tetrafluorophenol ester. The resulting Fe-*N*-suc-Df-TFP ester was reacted with bevacizumab under basic conditions (pH 9.5 to 9.7) for 30 min at room temperature. Subsequently, iron was removed using an excess of ethylenediaminetetraacetic acid (EDTA) for 30 min at 35°C, and the resulting *N*-suc-Df-bevacizumab was purified over a e and radiolabeled with ^89^Zr for 60 min in HEPES buffer at room temperature. Finally, ^89^Zr-*N*-suc-Df-bevacizumab was purified over a PD-10 column using 5 mg/mL gentisic acid in 0.9% NaCl (pH 4.9 to 5.4). The mean labeling efficiency was 71.1% ± 5.3%. The product was formulated and filter sterilized to 5 mg bevacizumab and 37 MBq Zr-89 per patient injection. These procedures resulted in a sterile final product with endotoxin levels <2.5 EU/mL. The radiochemical purity was >97% according to iTLC and HPLC, and the immunoreactivity as determined by an ELISA assay was >75%, which is optimal for this assay.

### PET scanning

All patients underwent a routine FDG PET/CT scan within 4 weeks prior to starting therapy. One week prior to therapy, the patients were injected intravenously with 36.4 ± 0.9 MBq ^89^Zr-bevacizumab, and 10-min static PET/CT scans of the thoracic NSCLC lesion were performed on days 4 and 7 post-injection. These parameters were based on previous ^89^Zr-trastuzumab analysis, showing appropriate doses and timing for visualization and quantification of uptake in HER2-positive tumors to be 37 MBq and days 4 to 5 after injection, respectively [20].

The scans were performed on a Gemini TF-64 PET/CT scanner (Philips Medical Systems, Cleveland, USA). PET data were normalized, and all appropriate corrections were applied for dead time, decay, randoms, and scatter. Reconstruction of PET data was performed using the BLOB-OS-TF reconstruction algorithm with CT-based attenuation correction, resulting in a final voxel size of 4 × 4 × 4 mm^3^, matrix of 144 × 144 × 45, and a spatial resolution of 5 to 7 mm full-width at half maximum [21]. Additional smoothing was performed, as this was shown to optimize quantitative accuracy and harmonized image quality [22].

### ^89^Zr-bevacizumab uptake analysis

FDG PET/CT scans were used to identify the exact location of the tumors. The PET data were analyzed using in-house-developed analysis tools within the IDL environment (Interactive Data Language Virtual Machine 6.2, RSI Inc., Boulder, CO, USA). Volumes of interest (VOI) were drawn manually on the CT images around the contours of the primary tumor (PT) and, if present, lymph node metastases (LNM) and non-lymph node metastases (NLNM). Additionally, VOI were drawn within the descending aorta (AD) and non-tumor tissue muscle (M), healthy lung (HL), and fatty tissue (FT). Standardized uptake value (SUV) parameters were calculated by normalizing VOI activity concentrations to injected dose and patient weight: SUV_peak_ (spheric VOI of 1.2-cm diameter positioned around the voxel with the highest uptake) and SUV_mean_ (mean activity in VOI/cc) from the AD VOI to calculate the tissue-to-blood ratio (TBR). SUV_peak_ and TBR for the abovementioned VOI were then compared for the scans at days 4 and 7.

### Statistical analysis

Statistical analysis was performed using SPSS software (SPSS for Windows 15.0, SPSS, Inc.). Correlations were explored using Spearman’s correlation coefficient (*r*_*s*_). A two-tailed probability value of *P* < 0.05 was considered significant. Values in the text and tables are presented as mean ± standard deviation (SD) unless stated otherwise.

## Results

**Figure 1:**
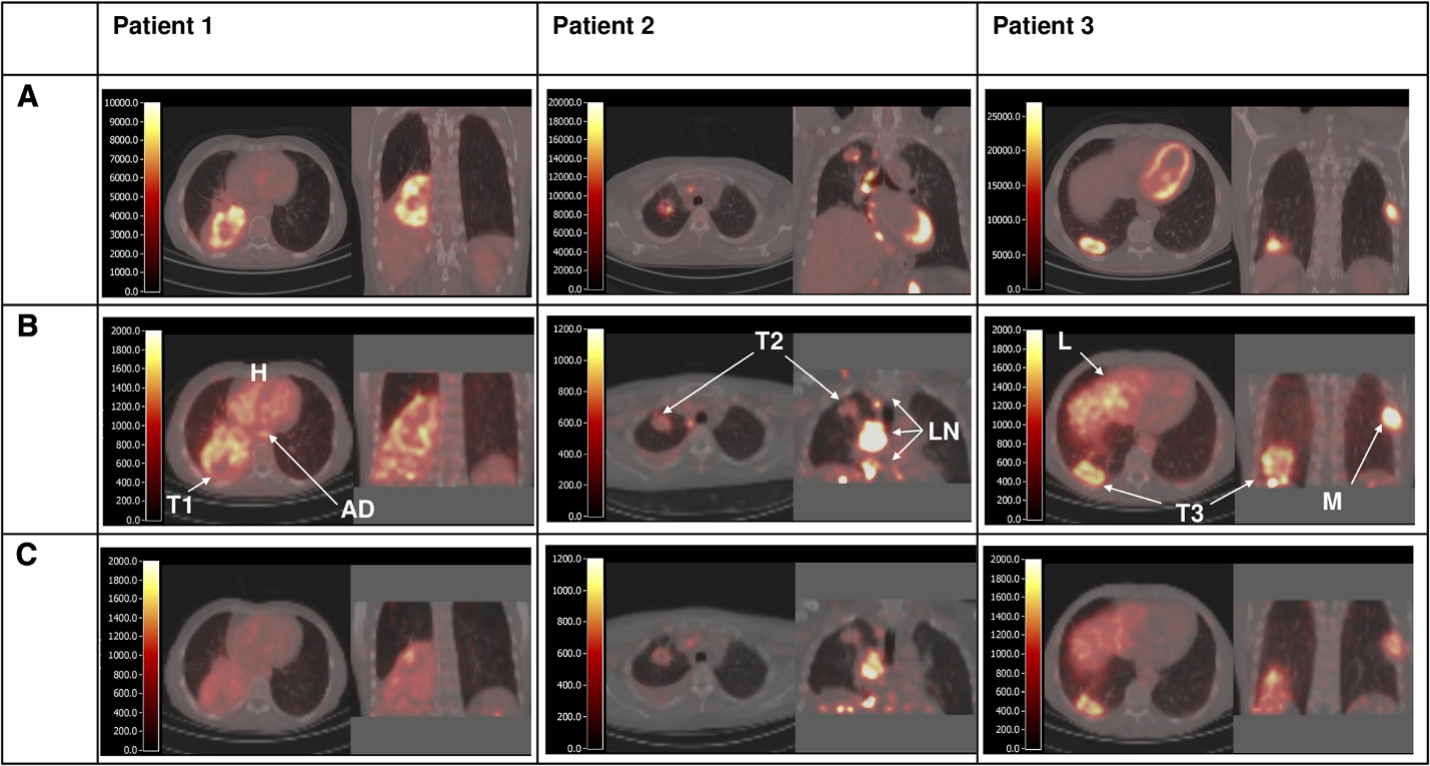
**Fused PET/CT images using FDG (A) and**^**89**^**Zr-bevacizumab at day 4 (B) and day 7 (C).** Per scan, an axial and coronal slice is shown. The color scale, indicating Becquerels per milliliter (BQML), ranges from 0 to a maximum value that corresponds with a SUV value of 6. In patient 1, a large tumor in the right lower lobe is seen. There is increased FDG uptake in the outer rims of the tumor and reduced uptake in the center of the tumor (probably due to necrosis). The ^89^Zr-bevacizumab image on day 4 also shows high uptake in the outer rims of the tumor (T1) and high blood activity concentration (aorta descendens (AD) and heart chambers (H)). Low uptake is found in non-tumor tissues, such as healthy lung, fat, and muscle. The ^89^Zr-bevacizumab image on day 7 shows high uptake in the outer rims of the tumor but low uptake in healthy tissues as well as low blood activity concentrations. In patient 2, the FDG scan shows increased uptake in both the primary tumor in the right upper lobe and enlarged mediastinal lymph node metastases. Interestingly, the ^89^Zr-bevacizumab images only show increased uptake in the lymph node metastases (LN), while the uptake in the primary tumor (T2) on both days 4 and 7 is faint. In patient 3, the primary tumor, the mediastinal lymph node metastases, and rib metastasis all show increased FDG uptake. The ^89^Zr-bevacizumab scans on days 4 and 7 show the highest uptake in the rib metastasis (M) and the primary tumor (T3), and moderate to high uptake is seen in the liver (L).

Seven patients were included in this study. The patient characteristics are summarized in Table 1. Nine mediastinal LNM were identified using FDG PET/CT scans within the thoracic field of view. Six patients showed NLNM. All tumor lesions showed visible ^89^Zr-bevacizumab uptake. To illustrate the findings as reported below, typical examples of fused PET/CT images using FDG and ^89^Zr-bevacizumab in three NSCLC patients are provided in Figure 1.

**Table 1:** **Patient characteristics**

Number	Sex	Age (years)	Primary tumor	Evaluable metastasis	Histology	Bevacizumab maintenance therapy	Best response	Site of progression	PFS (weeks)	OS (weeks)
1	F	56	RLL	-	AC	Yes	PR	LLL	56	79
2	M	51	RUL	Peritoneum	AC	Yes	PR	PC	13	17
3	M	59	RLL	Bone	AC	Yes	PR	RUL^a^	27	48
4	M	61	RLL	Bone	LCC	Yes	CR	RA	35	63
5	F	54	RLL	Bone	AC	Yes	PR	Brain	54	74
6	F	69	RH	Pericardium	AC	Yes	PR	MC	34	60
7	M	51	LH	Bone	AC	No	PD	Liver^a^	3	3

Evaluable metastases are non-primary lesions, located within the PET field of view. All patients received bevacizumab maintenance therapy, except for one patient, who died before bevacizumab maintenance therapy could be administered. The best tumor response to chemotherapy is shown. Progressive disease was seen in all patients. ^a^Two patients developed clinical disease progression before radiological progression. One patient developed progression due to brain metastasis. Sites of radiological progression are shown. PFS and OS were calculated in weeks, between start of chemotherapy and date of progression and death, respectively. AC, adenocarcinoma; CR, complete response; F, female; LCC, large cell carcinoma; LH, left hilum; LLL, left lower lobe; M, male; MC, meningitis carcinomatosa; OS, overall survival; PC, peritonitis carcinomatosa; PD, progressive disease; PFS, progression-free survival; PR, partial response; RA right adrenal gland; RH, right hilum; RLL, right lower lobe; RUL, right upper lobe; SDC, salivary duct carcinoma.

**Figure 2:**
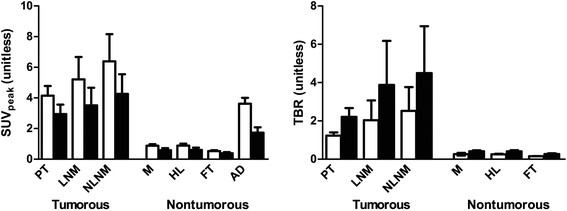
**Comparison of**^**89**^**Zr-bevacizumab SUV**_**peak**_**and TBR in different tissues.** The values were compared at day 4 (open bars) and day 7 (black bars). AD, aorta descendens; FT, fatty tissue; HL, healthy lung; LNM, lymph node metastasis; M, muscle; NLNM, non-lymph node metastasis; PT, primary tumor; TBR, tumor-to-blood ratio.

In all tumorous tissues (PT, LNM, and NLNM), ^89^Zr-bevacizumab uptake (SUV_peak_) was approximately four times higher than those in non-tumorous tissues (M, HL, and FT) on days 4 and 7, as shown in Table 2. For tumorous tissues, the SUV_peak_ in NLNM and LNM was approximately 54% and 26% higher as compared to that in PT on day 4, and 49% and 19% higher on day 7, respectively (see Figure 2).

**Table 2:** ^**89**^ **Zr-bevacizumab uptake parameters**

	Tumorous tissue			Blood (AD)	Non-tumorous tissue		
	PT	LNM	NLNM		Muscle	Fat	Lung
Day 4							
SUV_peak_	4.3	4.0	5.0	3.9	1.0	0.6	0.8
	(2.1 to 6.9)	(2.1 to 12.1)	(2.9 to 14.5)	(1.7 to 4.9)	(0.4 to 1.1)	(0.3 to 0.7)	(0.5 to 1.4)
TBR	1.2	1.1	1.2		0.3	0.2	0.3
	(0.7 to 1.8)	(0.8 to 7.2)	(0.7 to 8.6)		(0.1 to 0.3)	(0.1 to 0.2)	(0.2 to 0.3)
Day 7							
SUV_peak_	3.3	2.7	3.9	1.4	0.5	0.4	0.4
	(0.7 to 4.9)	(1.0 to 9.0)	(1.1 to 9.7)	(0.9 to 3.5)	(0.2 to 1.3)	(0.1 to 0.6)	(0.2 to 1.1)
TBR	0.8	1.6	1.9		0.5	0.3	0.4
	(1.0 to 4.0)	(1.1 to 15.3)	(0.9 to 16.5)		(0.3 to 0.6)	(0.1 to 0.4)	(0.2 to 0.6)

AD, aorta descendens; LNM, lymph node metastases; NLNM, non-lymph node metastases; PT, primary tumor; TBR, tumor-to-blood ratio. Median (range) is shown.

Remarkably, on day 4, activity concentration in blood (AD) was approximately the same as that in the PT, while the healthy tissue activity was clearly lower than that in PT (see Table 2 and Figures 1 and 2). However, between days 4 and 7, tumor TBR increased from 1.23 ± 0.4 to 2.2 ± 1.2.

**Figure 3:**
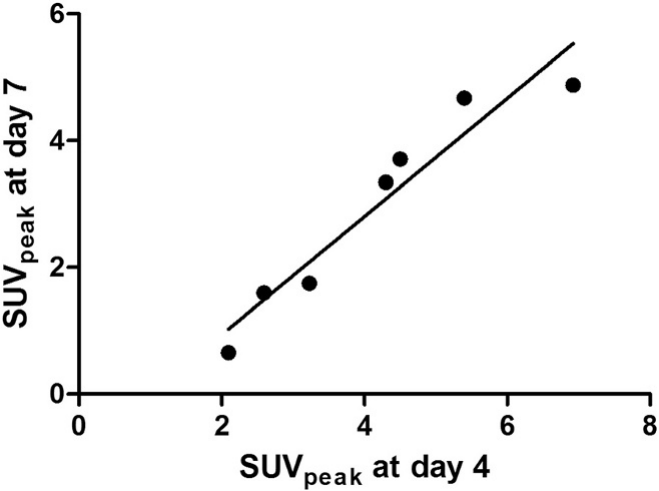
**Comparison of tumor**^**89**^**Zr-bevacizumab uptake (SUV**_**peak**_**) on days 4 and 7.** A correlation coefficient (*r*_*s*_) of 1 (*P* < 0.001) was found.

SUV_peak_ correlated strongly between days 4 and 7, as indicated by an *r*_*s*_ of 1 (*P* < 0.001). Figure 3 shows the correlation between days 4 and 7 for SUV_peak_.

**Figure 4:**
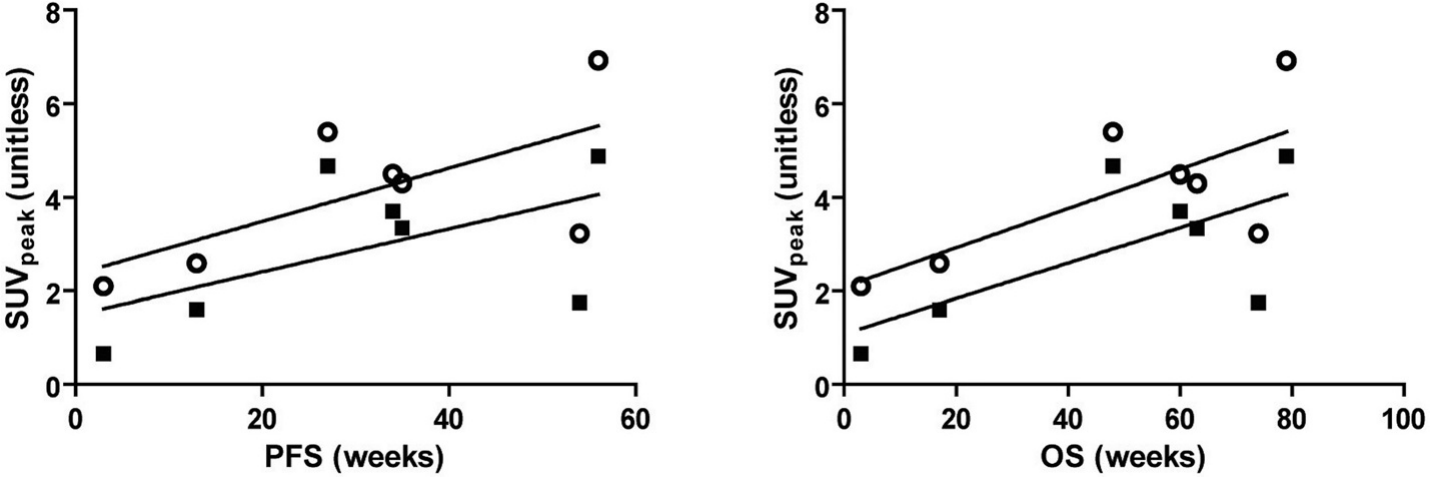
**Correlations between SUV**_**peak**_**and clinical outcome (PFS and OS).** The correlations are for day 4 (open circles) and day 7 (black squares). Correlation coefficients were not statistically significant for both PFS and OS, with *r*_*s*_ of 0.64 (*P* = 0.139) at days 4 and 7, respectively. PFS, progression-free survival; OS, overall survival.

As shown in Figure 4, correlations between tumor SUV_peak_ and outcome parameters showed a positive trend, but did not reach statistical significance. SUV_peak_ showed weak correlation with both PFS and OS on days 4 and 7. (*r*_*s*_ was 0.64 with a *P* = 0.139 for both PFS and OS on days 4 and 7, respectively).

## Discussion

### Visualization of tumor lesions

Prior to this study, it was unclear whether NSCLC tumors could be imaged by ^89^Zr-bevacizumab. The results of this pilot study show that all tumor lesions (PT, LNM, and NLNM) had a higher ^89^Zr-bevacizumab uptake as compared to non-tumor background tissues, allowing for visualization and analysis of tumor ^89^Zr-bevacizumab uptake.

This increased uptake may be caused by accumulation of VEGF in the tumor, due to high paracrine expression and subsequent binding to extracellular matrix glycoproteins such as heparan sulfate proteoglycans (HSPG) and neuropilins (NRP). These glycoproteins act as non-signaling co-receptors that facilitate binding of VEGF to VEGFR molecules [6]. Another mechanism that may contribute is the internalization of ^89^Zr-bevacizumab into cells within the tumor. After internalization, the ^89^Zr label may become trapped in the lysosomes and show up on the PET scan [23].

Our findings are in accordance with the limited number of publications of previous preclinical and clinical data showing that high-VEGF-A-expressing tumors are associated with high tumor-to-background ^89^Zr-bevacizumab uptake. Nagengast et al. [19],[24] showed that high-VEGF-producing SKOV-3 ovarian tumor xenografts had a higher uptake of ^89^Zr-bevacizumab than of ^89^Zr-labeled IgG, which served as a control. In a follow-up study, the same researchers found that ^89^Zr-bevacizumab uptake decreased in (NVP-AUY922 sensitive) A2780 ovarian tumor xenografts after 2 weeks of antitumor therapy (using NVP-AUY922), while in the therapy-resistant CP70 xenografts, ^89^Zr-bevacizumab uptake did not change. van der Bilt et al. [25] showed a decrease in ^89^Zr-bevacizumab uptake in A2780 ovarian tumor xenografts after 2 weeks of everolimus therapy, while ^89^Zr-bevacizumab uptake remained unchanged in other organs, matching *ex vivo* measures of VEGF-A levels [19],[24],[25]. Recently, a first clinical study using ^89^Zr-bevacizumab was reported. In breast cancer patients, tumors with elevated VEGF-A levels showed higher ^89^Zr-bevacizumab uptake than the background of healthy breast tissue [26].

We found that tumor-to-background ratios were high at days 4 and 7; however, tumor-to-blood ratios were higher on day 7, due to a relative decrease of blood activity concentrations as compared to tumor activity concentrations. However, image quality at day 7 was hampered due to physical decay of the tracer. The optimal time for ^89^Zr-bevacizumab seems to be between 4 and 7 days post-injection.

Lymph node metastases and especially NLNM showed higher uptake than pulmonary lesions. This may be caused by the fact that pulmonary lesions suffered more from breathing movement-induced partial volume effects than LNM and NLNM, which were relatively fixed to bone and peritoneum. Although all tumoral lesions were visible in this study, the quantification of uptake in small-volume lesions needs to be interpreted with caution, as these tumors suffer even more from this partial-volume-induced underestimation of tracer uptake.

### Clinical outcome

Although our results did not show a significant correlation between tracer uptake and PFS, a positive trend was observed. As tumor ^89^Zr-bevacizumab uptake may represent the level of tumor VEGF, this technique might offer a predictive biomarker for bevacizumab treatment efficacy. At present, there is an absence of clinically useful predictive biomarkers. For example, several blood biomarkers, such as VEGF-A and NRP-1 using newly developed sensitive assays, have been proposed to predict bevacizumab treatment efficacy, but their value is still the subject of debate [6],[27].

### Limitations

As this was a pilot study, only a limited number of patients were included. However, the results obtained were consistent for all patients, indicating that larger clinical trials are warranted.

Another limitation was the absence of arterial blood sampling for blood and plasma activity and metabolite analysis, which could have provided a more accurate quantification of tracer uptake. However, because patients already underwent several PET/CT scans, additional blood sampling for research purposes was considered too high a burden. Furthermore, previously published data show a good correlation between image-derived activity from ^89^Zr-labeled antibodies and blood activity [15],[17]. Additionally, ^89^Zr-bevacizumab was found to be highly stable in plasma, as only a 6% decrease in protein-bound radioactivity was seen after 168 h (stored in serum at 37°C) [19]. Although NSCLC was initially diagnosed in all patients, no extra tumor biopsy was taken prior to scanning for VEGF-A staining, again because this was considered too burdensome.

### Future perspectives

The results of this study show tumor specific uptake of ^89^Zr-bevacizumab. Future studies should consider not to include small lesions that could suffer from partial volume effects. Furthermore, the optimal timing should be investigated, as this can be expected to be between day 4 and day 7 post-injection. To better understand the physiological processes causing tracer accumulation, concurrent pathology data (e.g., angioproliferative markers) should be assessed by taking biopsies prior to scanning. Quantification of uptake may be improved by using blood sampling for plasma radioactivity assessment.

The effect of perfusion on ^89^Zr-bevacizumab should be analyzed. In this study, an assessment of tumor blood perfusion, e.g., with [^15^O]H_2_O PET scan, was not performed. Using [^15^O]H_2_O PET scans, van der Veldt et al. [28] showed that the intratumoral distribution of docetaxel followed the tumor perfusion patterns. Therefore, tumor perfusion data may have provided additional understanding of the distribution of tumoral ^89^Zr-bevacizumab uptake.

^89^Zr-bevacizumab PET may be used as an imaging agent, as tracer uptake showed a trend towards a positive correlation with PFS and OS. It should be noted that patients were treated with carboplatin-paclitaxel-bevacizumab (CPB) followed by bevacizumab maintenance therapy. PFS was the result of both CPB therapy and bevacizumab. In future studies, ^89^Zr-bevacizumab scans should ideally be performed at two time points, i.e., prior to CPB therapy and also prior to bevacizumab maintenance therapy. This is because the post-CPB-altered tumor VEGF status is unknown; however, this new post-CPB VEGF status may be a better predictor for sensitivity to subsequent bevacizumab maintenance therapy.

## Conclusions

This pilot study demonstrates that ^89^Zr-bevacizumab PET imaging in tumors is feasible. Larger studies are needed to validate and substantiate these findings. ^89^Zr-bevacizumab PET merits further investigation, aiming to evaluate its use as a predictive imaging biomarker.

## Authors’ original submitted files for images

Below are the links to the authors’ original submitted files for images. Authors’ original file for figure 1 Authors’ original file for figure 2 Authors’ original file for figure 3 Authors’ original file for figure 4

## Abbreviations

AD: aorta descendens
CPB: carboplatin-paclitaxel-bevacizumab
CT: computed tomography
FDG: fluorodeoxyglucose
FT: fatty tissue
HL: healthy lung
LNM: lymph node metastasis
M: muscle
MBq: megabecquerels
NLNM: non-lymph node metastasis
NRP: neuropilin
NSCLC: non-small cell lung cancer
OS: overall survival
PET: positron emission tomography
PFS: progression-free survival
PT: primary tumor
SUV: standardized uptake value
TBR: tumor-to-blood ratio
VEGF: vascular endothelial growth factor
VOI: volume of interest
WHO: World Health Organization
Zr: zirconium
