# Supplementary figures and images for: Pilot study of 89Zr-bevacizumab positron emission tomography in patients with advanced non-small cell lung cancer
Source: EJNMMI Res. 2014 Aug 2;4:35. doi: 10.1186/s13550-014-0035-5 (PMC4884046; doi:10.1186/s13550-014-0035-5)

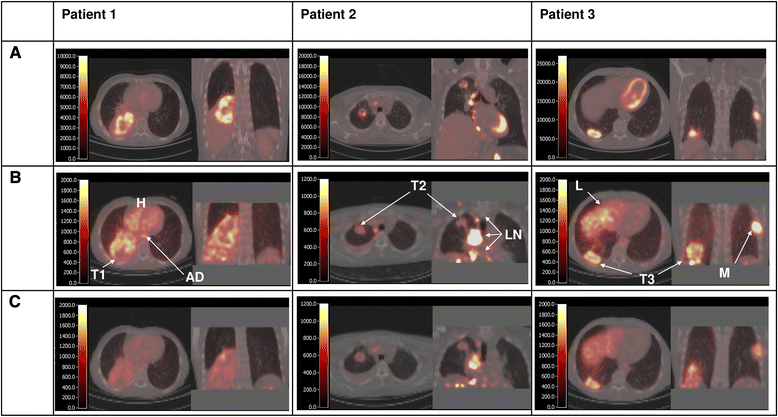

Supplement: Supplementary file 1 — Authors’ original file for figure 1 [file 13550_2014_35_MOESM1_ESM.gif]

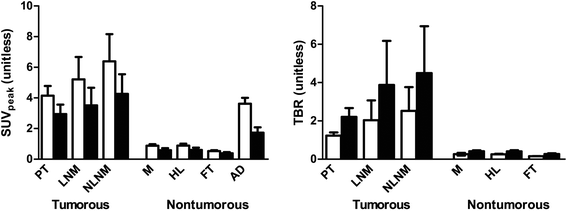

Supplement: Supplementary file 2 — Authors’ original file for figure 2 [file 13550_2014_35_MOESM2_ESM.gif]

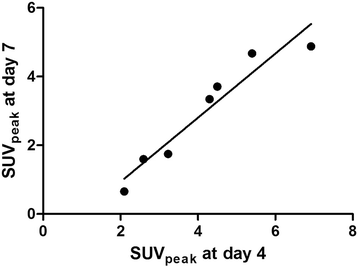

Supplement: Supplementary file 3 — Authors’ original file for figure 3 [file 13550_2014_35_MOESM3_ESM.gif]

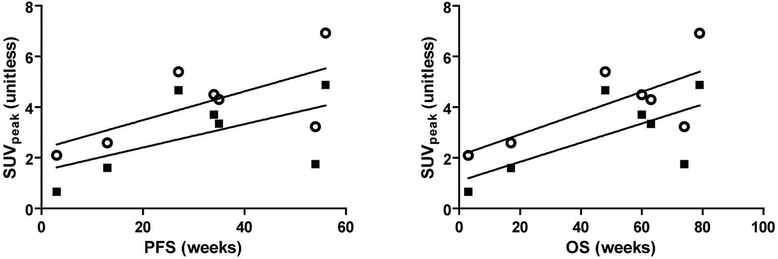

Supplement: Supplementary file 4 — Authors’ original file for figure 4 [file 13550_2014_35_MOESM4_ESM.gif]
